# A study on plant root apex morphology as a model for soft robots moving in soil

**DOI:** 10.1371/journal.pone.0197411

**Published:** 2018-06-06

**Authors:** Anand Kumar Mishra, Francesca Tramacere, Roberto Guarino, Nicola Maria Pugno, Barbara Mazzolai

**Affiliations:** 1 Center for Micro-BioRobotics, Istituto Italiano di Tecnologia, Pontedera, Italy; 2 The BioRobotics Institute, Scuola Superiore Sant’Anna, Pontedera, Italy; 3 Laboratory of Bio-Inspired & Graphene Nanomechanics, Department of Civil, Environmental and Mechanical Engineering, University of Trento, Trento, Italy; 4 Ket Lab, Edoardo Amaldi Foundation, Italian Space Agency, Rome, Italy; 5 School of Engineering and Materials Science, Queen Mary University of London, London, United Kingdom; University of Vermont, UNITED STATES

## Abstract

Plants use many strategies to move efficiently in soil, such as growth from the tip, tropic movements, and morphological changes. In this paper, we propose a method to translate morphological features of *Zea mays* roots into a new design of soft robots that will be able to move in soil. The method relies on image processing and curve fitting techniques to extract the profile of *Z*. *mays* primary root. We implemented an analytic translation of the root profile in a 3D model (CAD) to fabricate root-like probes by means of 3D printing technology. Then, we carried out a comparative analysis among the artificial root-like probe and probes with different tip shapes (cylindrical, conical, elliptical, and parabolic) and diameters (11, 9, 7, 5, and 3 mm). The results showed that the energy consumption and the penetration force of the bioinspired probe are better with respect to the other shapes for all the diameters of the developed probes. For 100 mm of penetration depth and 7 mm of probe diameter, the energy consumption of the bioinspired probe is 89% lesser with respect to the cylindrical probe and 26% lesser with respect to the conical probe. The penetration performance of the considered tip shapes was evaluated also by means of numerical simulations, obtaining a good agreement with the experimental results. Additional investigations on plant root morphology, movement strategies, and material properties can allow the development of innovative bioinspired solutions exploitable in challenging environments. This research can bring to breakthrough scenarios in different fields, such as exploration tasks, environmental monitoring, geotechnical studies, and medical applications.

## Introduction

Soil exploration is one of the activities most practiced by humans for natural resources research, civil construction, and geotechnical study. For addressing such issues, different ways are being used, including: (i) drilling, (ii) excavation, (iii) subsurface sounding tests, and (iv) geophysical methods [1, 2]. Drilling (i) is a perforation process that exploits a motor (heat, hydraulic, pneumatic or electric motor) for pushing a drill bit (cutter) into the soil and making a hole. It is the most used method for extraction of water, petroleum, and natural gases; its application is so wide that it has been extended to the space exploration [3–6]. The second method, excavation (ii), is an ancient method used for making wells by means of hoes, shovels, earthmovers, etc. It is employed for water harvesting and building construction, but also for archaeological studies and soil inspection [1, 2, 7]. The remaining two methods, namely subsurface sounding tests (iii) and geophysical methods (iv), are mainly exploited in geotechnical studies. Subsurface sounding tests (iii) allow measuring soil strength and explore soil profile by pushing a probe into the soil. The typical probing tests are Standard Penetration Test (SPT) and Cone Penetration Test (CPT). In SPT, a thick-walled sample tube is driven into the ground at the bottom of a borehole by blows from a slide hammer; whereas, in CPT, a cone penetrometer is pushed into the soil by applying, from the top, static (hammer blow) or dynamic loads (hydraulic actuators) [1, 8–13]. The last exploration solution, i.e., geophysical methods (iv), is a family of solutions used to study the soil and subsoil profile. The most exploited techniques are based on seismic reflection and electro-resistivity measurements. In the first case, the reflection response of controlled seismic waves is detected to get information about the earth's subsurface properties. In the second technique, the electrical resistivity of soil is measured for imaging sub-surface structures [14–19]. Although all the four above mentioned exploration techniques are widely used, they present some limitations. The main important limitations are: the most successful penetration method (drilling) requires high energetic costs, bulky infrastructure, and high impact on natural environments (not only soil destruction but also damage to surrounding natural areas); the less invasive measurement techniques (geophysical methods) present several drawbacks (e.g., in the electrical resistivity method, the reliability is affected by metal pipes, electrical conductors, and sloping of strata); in the case of seismic survey instead, it is quite accurate for horizontal layers structure but requires large source-receiver offsets [1, 3].

Looking at nature, there are many living beings, such as earthworms, wasps, clams, siphons, moles, etc., that can perform penetration and perception tasks in soil [3, 5, 20–24]. Among digging organisms, plants are particularly efficient in terms of perforation performance (1 MPa axial stress) and are able to reach considerable depths (up to 100 m [25]). The key features that make plant roots so efficient are: (i) minimal friction strategy, (ii) crack propagation exploitation, and (iii) high structural stability. (i) Plants adopt an efficient strategy for moving in soil based on a growth from the tip. In fact, plant roots overcome and survive into high frictional and abrasive soil environment by pushing only the apical part while the whole structure remains stationary and in contact with the surrounding medium (soil). This strategy, together with mucus exudation and sloughing cells release, allows plants to create low friction channels and increases the penetration efficiency [26–28]. In addition to such peculiarity, plants are also able (ii) to generate lateral expansion for making cracks and going through (reducing significantly the penetration efforts); and (iii) to improve the anchorage developing hairs and lateral roots [29–31].

The first attempt of taking inspiration from plant roots to conceive innovative digging artificial solutions is described in [32]. In this work, authors proposed a mechatronic system for soil exploration. The system can steer in all directions and can perform gravitropism and hydrotropism movements (the ability to follow the gravity and moisture, respectively). In [33], authors proposed a device inspired by the role of sloughing cells and root hairs in plants, demonstrating that this approach allows reducing penetration force by 30% in a granular soil medium. Further investigations on plant root mechanisms for soil penetration allowed the same authors to develop a plant-inspired robot able to grow in soil from its tip using an additive layer technique [34, 35]. This system, which implements the plant growth strategy, has shown a reduction in energy consumption by 70% with respect to an analogous artificial solution pushed in soil from the top. A study on the influence of probe’s morphology in penetration efficiency was presented by Tonazzini et al. [36]. Authors compared different shapes of tips in an artificial soil to select that better accomplishing penetration tasks. The results showed that a conical shape represents the best solution for penetration in glass bead environment. A further improvement, in the present work, we investigated the contribution of root-like probe morphology, especially of its apical region that represents the dynamic element of the system, in soil penetration. Specifically, we developed probes with different diameters and different shapes and compared their performance with a root-like probe in real soils, such as sandy loam. In summary, we here describe the method developed for extracting the root tip morphology, the fabrication technique used for making a bioinspired artificial prototype, discrete element simulations of probes with different shapes to correlate the experimental results, and the experimental setup used for evaluating achievable performance in soil penetration.

### Gross morphology of a plant root

**Fig 1:**
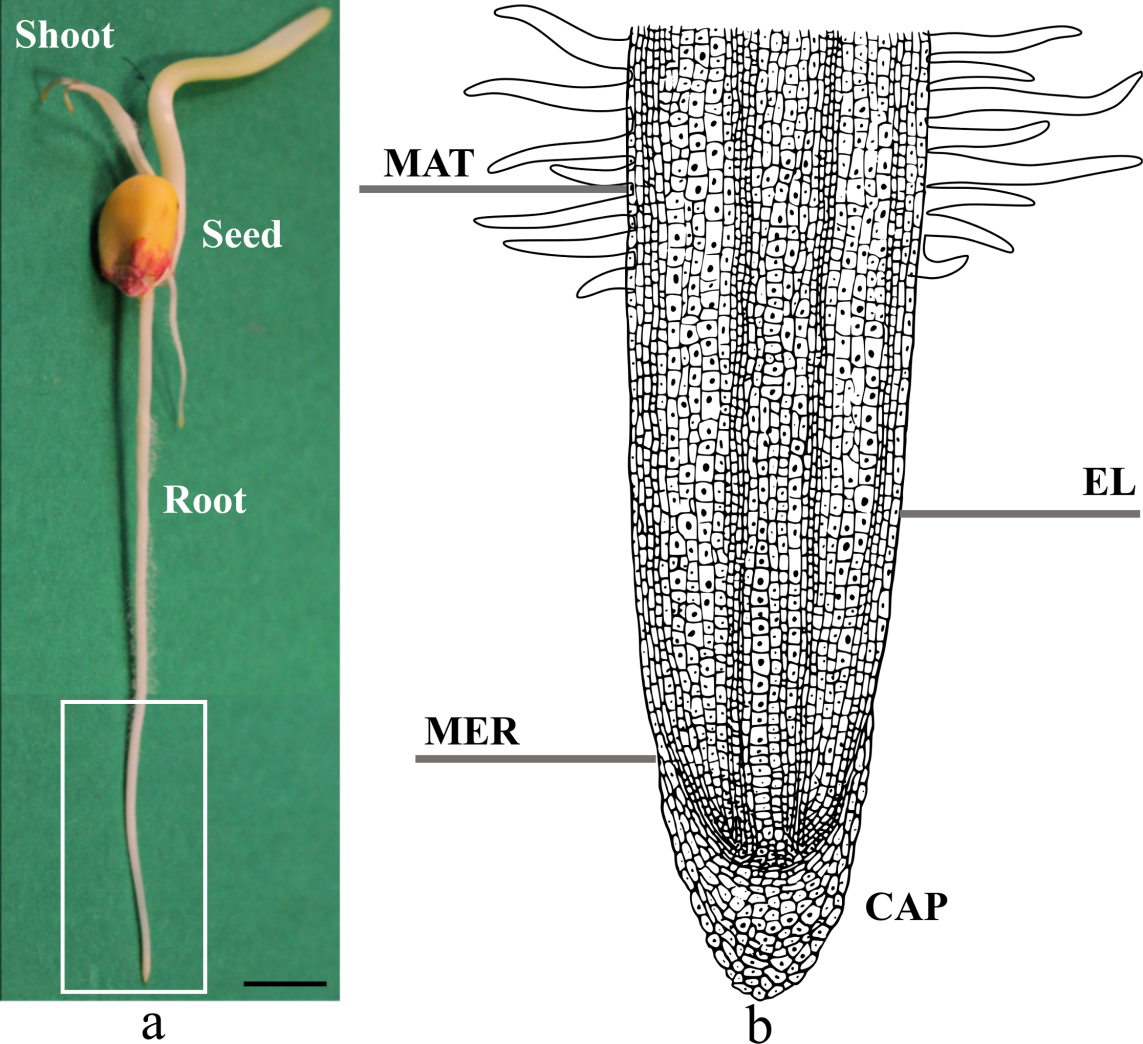
**a) 3-day old plant root.** Photograph of a *Zea mays* plant where it can be recognized the **seed,** the **shoot** (aerial part of the plant), and the primary **root** (underground part of the plant). The scale bar equals 10 mm. **b) Structure of the plant root.** Enlargement of the white box in (a). **MAT,** mature zone; **EL**, elongation zone; **MER,** meristematic zone; and **CAP,** root cap.

Root is a plant organ that generally lies below the surface of soil (Fig 1) and consists of: a root cap, and a meristematic, elongation, and mature zone. The root cap (CAP in Fig 1A) is the most apical part of a root and has a covering role in protecting the meristematic zone (described in the following) from abrasion phenomena, bacteria, and microorganisms. The cap is also fundamental for reducing the soil penetration resistance by releasing sloughing cells and mucilage exudation. The meristematic zone (MER in Fig 1B) is the root region where undifferentiated cells are located. Therefore, this portion is fundamental for providing new cells for root growth. The elongation zone (EL in Fig 1B) is located just above to the meristematic zone. In this region, cells divide and elongate up to ten times of their original size and play a fundamental role in crack propagation inside the soil (for *Zea mays;* axial pressure up to 1.4 MPa and radial pressure up to 0.6 MPa can be generated [29, 37]). The mature zone (MAT in Fig 1B) is a stationary root portion where all the cells are matured. It is characterized by the presence of lateral hairs and plays an important role in anchorage [34, 38].

## Material and methods

### Seed germination and microscope analysis

We used maize (*Zea mays*, wild type, Ver. Kubrick Societa’ Italiana Sementi S.p.A) seeds as a model in this study. The seeds were sterilized putting them in a flax with a solution of 50% of bleach and 50% of deionized water on magnetic stir for 15 minutes for two times. Afterwards, the seeds were covered with wet blotting papers and kept for 3 days in a growing chamber (Seed germinator SG 15, Nuova Criotecnica Amcota) without any obstacle at a temperature of 25°C and a humidity of 60% [39]. After 3 days of germination, we carried out a morphometric analysis (described in the following section) of root tip (N = 11) analysing the pictures captured with an optical digital microscope (KH-7700 HIROX with AD5040HIS lens).

### Morphometric analysis

**Fig 2:**
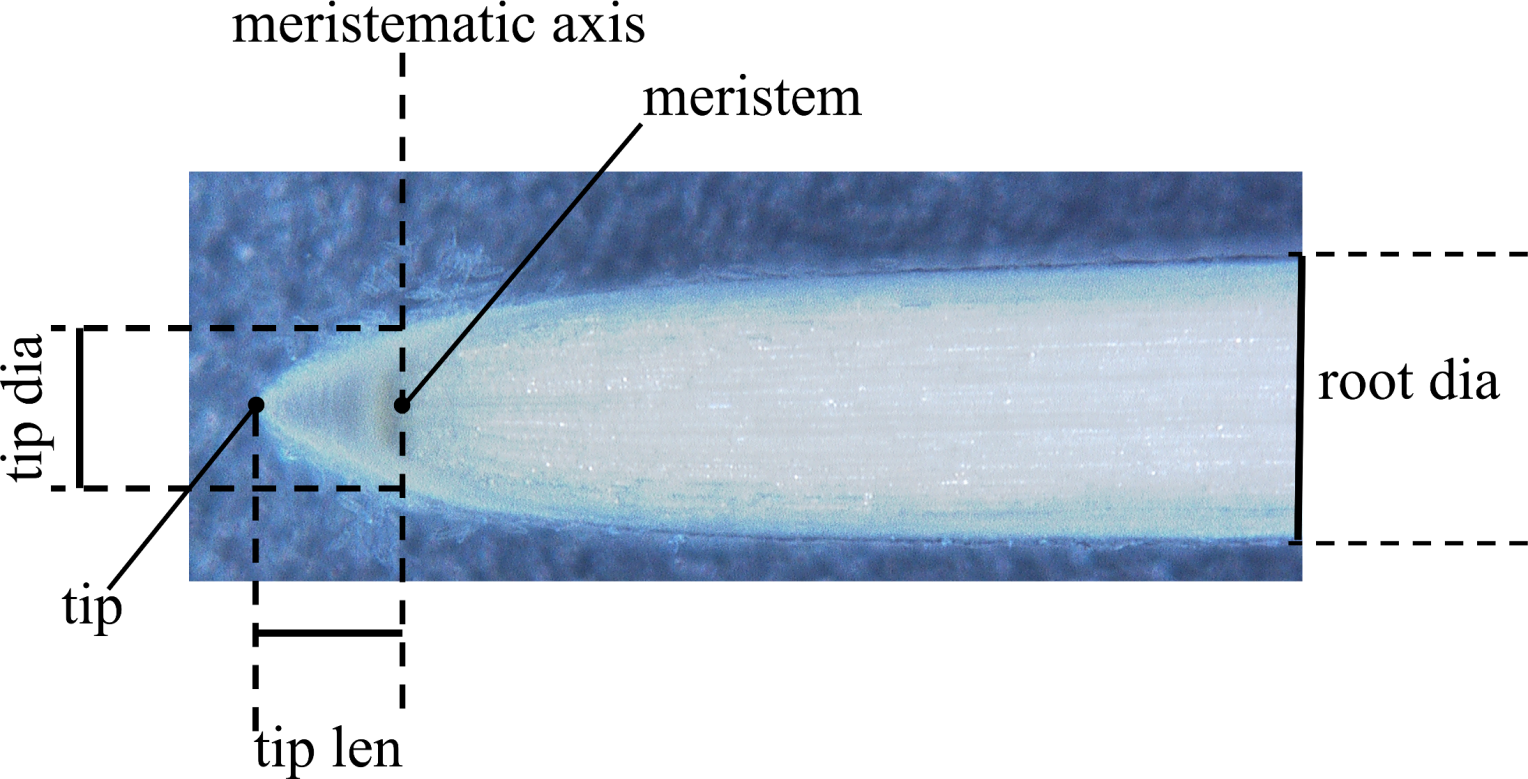
Morphometric analysis of a *Zea mays* root tip. *Meristematic axis* is the transversal axis that passes throughout the meristem, which is the root zone where growth takes place. *tip dia*, root tip diameter; *tip len*, root tip length; *root dia*, root diameter in correspondence of the mature region.

**Fig 3:**
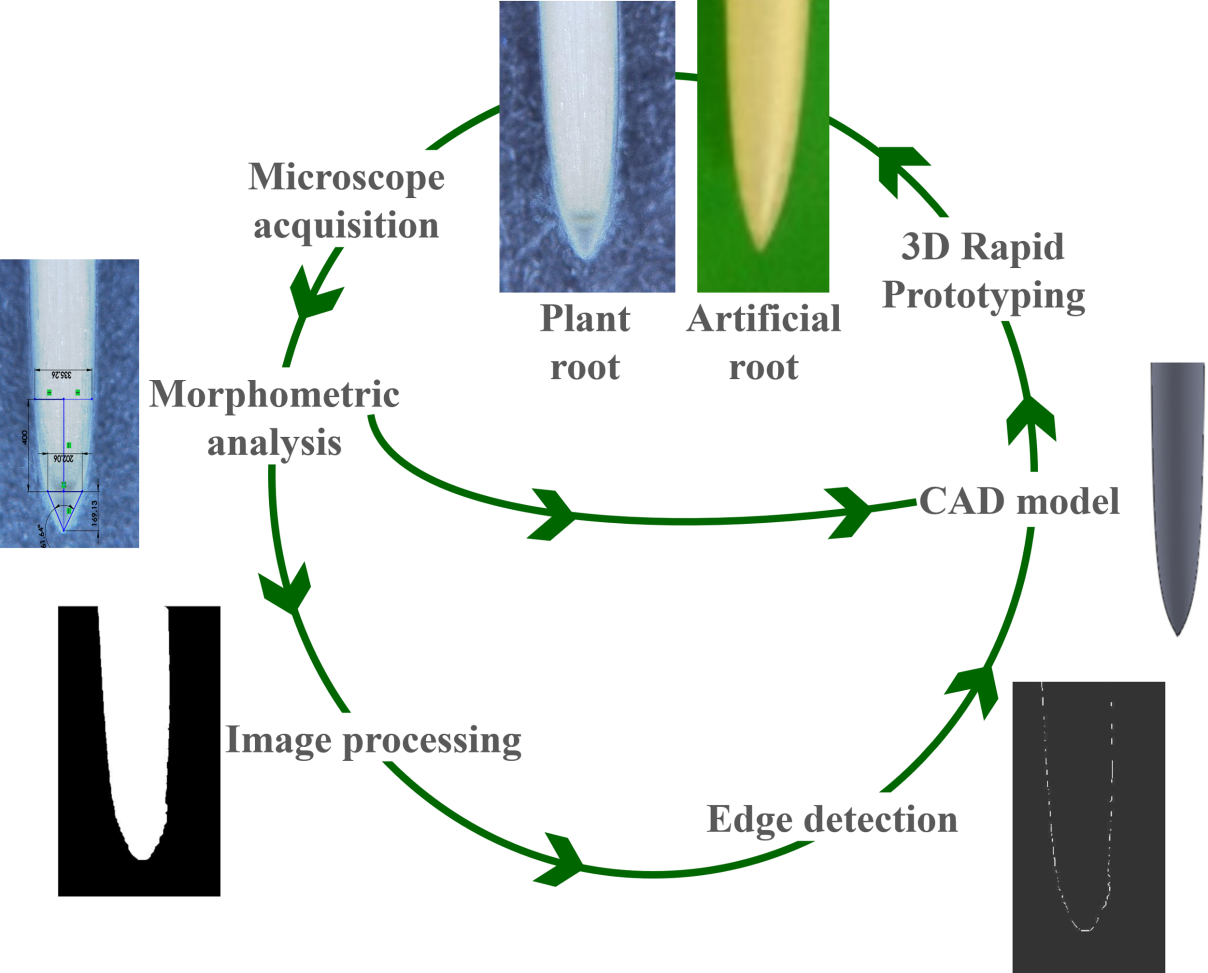
From a primary plant root to an artificial probe. Schematic view of the translation method from the natural system to the artefact. The root tip morphology was captured using an optical microscope (KH-7700 HIROX with AD5040HIS lens). Some crucial features were directly acquired from pictures (tip length and tip diameter); the remaining feature (tip profile) was extracted exploiting image processing and fitting techniques. The mathematical function that better follows the tip profile and the results of the morphometric analysis were taken into account to design the bioinspired probe. The probe was then fabricated in FullCure720 by 3D printing process.

We used a morphometric analysis to define two crucial features: the tip axial-transverse ratio and the tip profile. The first parameter refers to the ratio between the measured tip length and tip diameter (Fig 2). We detected the tip profile by image processing and fitting techniques (Fig 3). Then, we acquired the microscope images in MATLAB R2012a, Images Processing Toolbox release 2012a, MathWorks Inc., United States. They were first aligned and then processed with the image processing toolbox (i.e. background removal, image segmentation, and edge detection). This phase is fundamental for making root profile evident and proceeds with a fitting phase. Different mathematical functions were used to find the function that better follows the tip morphology, such as Gaussian, exponential, power, and polynomial functions.

### Design and fabrication of probes

#### Bioinspired probe

We used the morphology of *Zea mays* root tip for designing the model of a bioinspired probe in SolidWorks 2010. Then, we used computer aided design (CAD) model for making the artificial prototypes in FullCure720 (for details refer to [40]) by an additive manufacturing technology (3D printing) (Fig 3).

#### Other artificial probes

**Fig 4:**
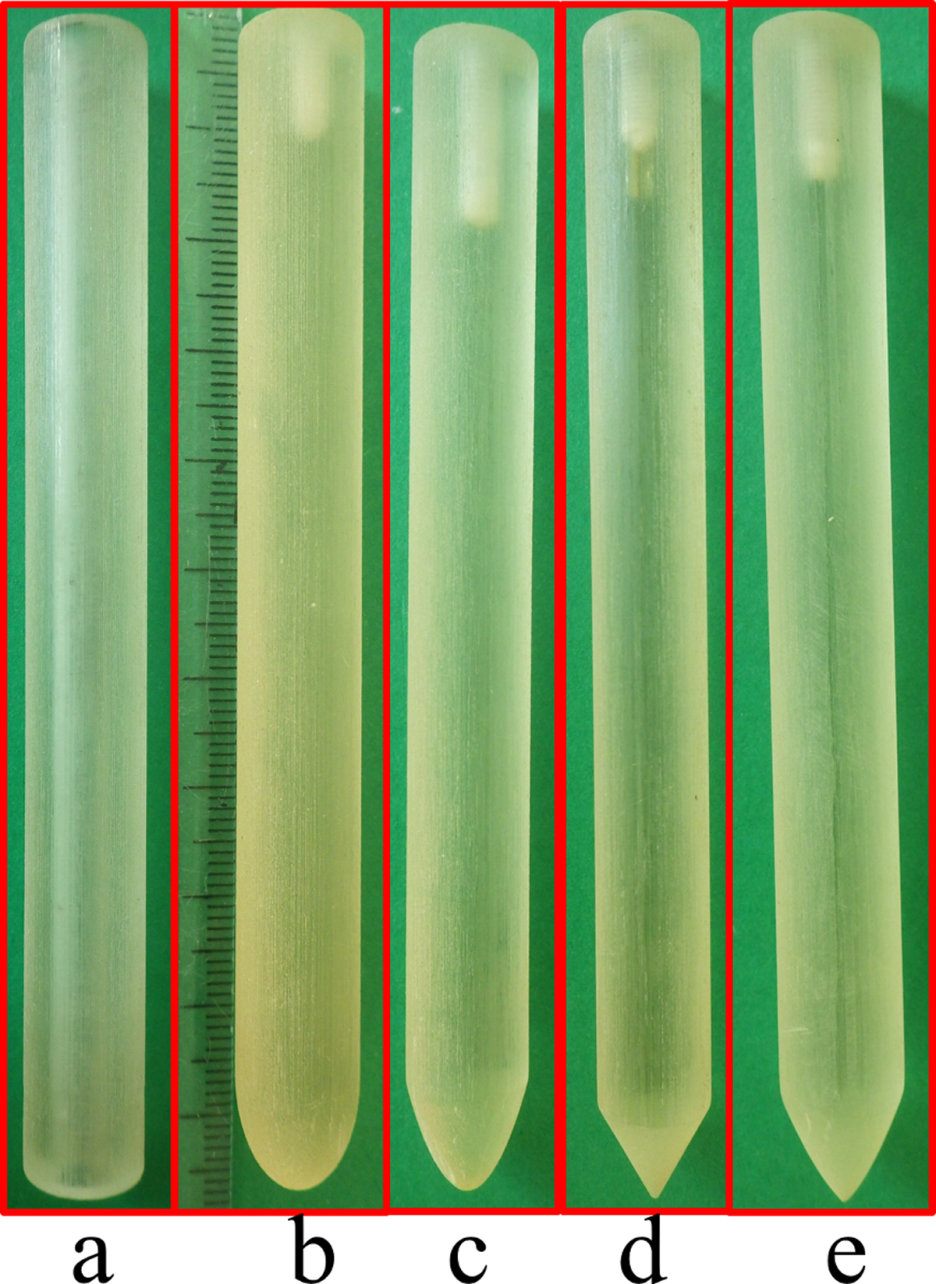
Artificial probes. Six different probes were fabricated (in the picture with 11 mm diameter) with (a) cylindrical; (b) elliptical; (c) parabolic; (d) conical; (e) and root-like.

In order to estimate soil penetration performance (force and expended work) of the bioinspired probe, we also manufactured other artificial probes by maintaining the same general features of the root (tip diameter and tip length) but modifying the standard tip profile (i.e. conical, cylindrical, parabolic, and elliptic) (Fig 4). The scale effect in soil perforation was quantified by comparing the performance of each artificial probe shape at different diameters (11, 9, 7, 5, and 3 mm). All the experimental data were analysed using ANOVA single factor method to evaluate possible differences among probes results.

### Protocol for penetration experiments

Penetration tests were carried out in soil by a Universal Testing Machine (UTM, Zwick/Roell Z005). We selected sandy loam soil and, before carrying out tests, it was kept under the sun to dry for two weeks, and then sieved to obtain homogeneous soil. After the sieving, the soil was characterized as sandy loam with 19.30% of silt, 12% clay, and 68.70% sand (see the S1 Fig). At this stage, it was kept inside an oven at 105°C for 10 h to remove moisture. The dried soil was put in an elliptical plastic container *D*_1_ × *D*_2_ × *H* (22x16x20 cm) for the penetration experiments. The bulk density of the obtained medium (1.56 g/cm^3^) was measured as follows:

$$d_{b}=\frac{W_{ds}}{V_{{}_{c}}}$$ (1)

Where *d*_*b*_ = soil bulk density, *W*_ds_ = weight of dried soil in the container, *V*_c_ = volume of the container occupied by soil. In order to avoid that the container size affects the results of the penetration tests, we always used a container diameter (*D*_*c*_) always more than 20 times ($\frac{D_{c}}{D_{p}}$>20) of the probe diameter (*D*_*p*_) [41].

**Fig 5:**
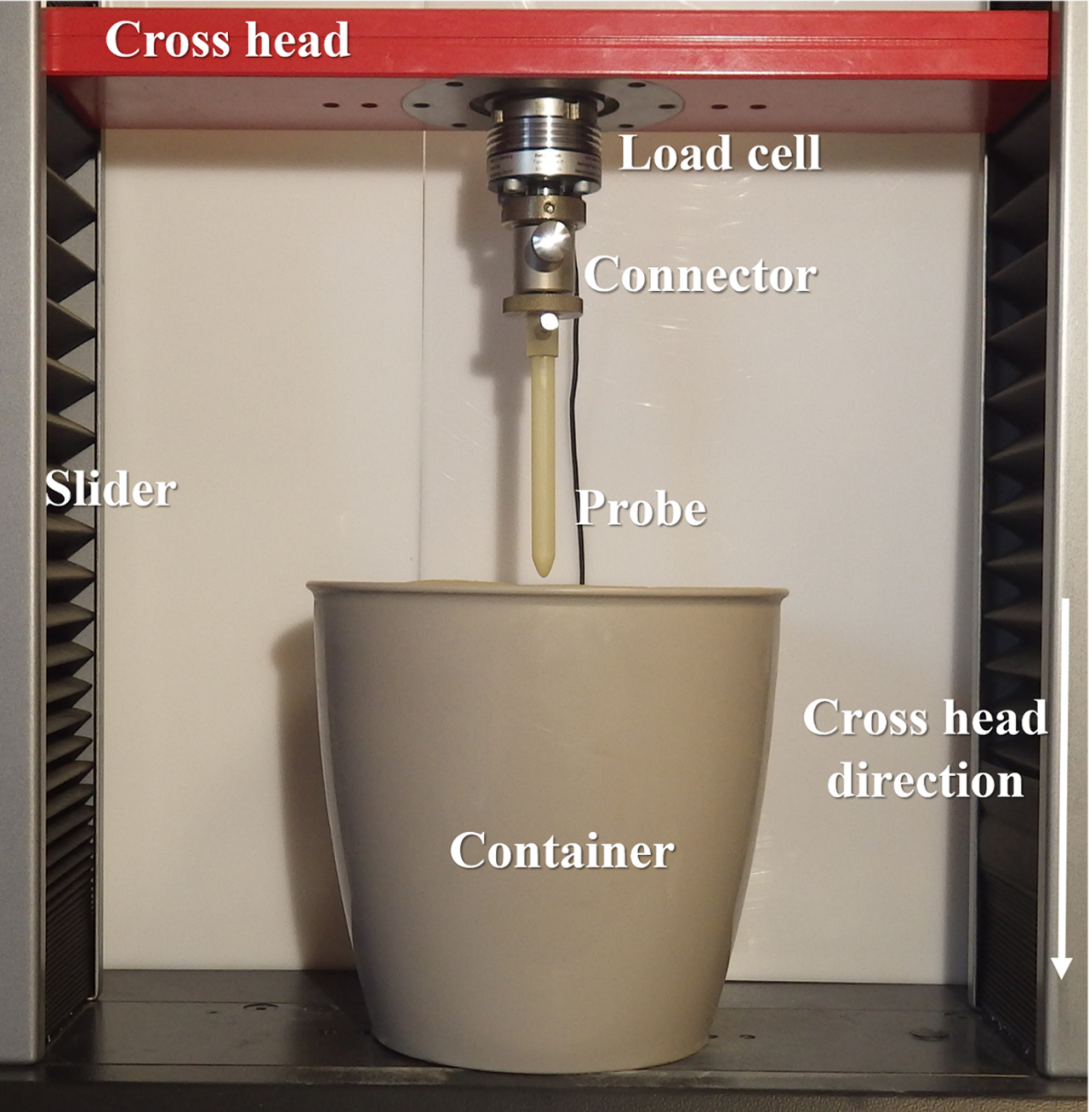
Experimental apparatus. The artificial probe is pushed from the top in soil using a UTM cross head.

During the experiments, all the developed probes were pushed in soil at 10 mm/min speed and up to 100 mm depth from their top using a UTM cross head. The resistance force was acquired by two types of load cells, ±1 kN and ±50 N with 0.001% of resolution. The probe was directly connected to load cell, where load cell was fixed on the cross head of UTM (Fig 5). The soil was taken out from the container and refilled [42] after each trial to maintain constant test conditions (impedance, consistency, etc.) and to avoid particle jamming pattern.

### Numerical simulations

Numerical simulations were carried out by employing the Discrete Element Method (DEM) [43]. The particles were randomly packed under the action of gravity in a cylindrical volume, constrained by frictional walls, while the probes were imported as Standard Triangulation Language (STL) meshes. The particle-particle and particle-wall collisions were modelled through a Hertz contact law [44]. Each simulation is composed of two distinct steps: the packing of the particles, until a quasi-stationary state is reached, and the subsequent penetration of the probe at a constant velocity. In addition, we carried out five simulations with different random starting positions of the particles, thus all the results are presented with an average value and a standard deviation.

DEM simulations of large systems are extremely expensive from the computational point of view, thus here only the probes with diameter *D*_*p*_ = 3 mm were simulated, in order to reduce the computational volume. For the same reason, the considered container is smaller than that used in experiments. Both choices, anyway, are not expected to affect the order relation among the different probe geometries. The effect of the probe diameter, in fact, as shown later for the experiments, has impact only on the absolute value of the forces, which scale roughly linearly with *D*_*p*_ (see the S12 Fig). The effect of the container walls, instead, is discussed in detail in the Supporting Information (see the S1 Note).

The soil was approximated with a monodisperse distribution of spherical particles with an arbitrary choice of their diameter *d*, with *d* << *D*_*p*_. The objective of the simulations carried out here, in fact, is to discriminate among the different probe shapes, whose order relation is not expected to change with the soil properties. Moreover, a similar DEM representation of soil has already been used in the literature, e.g. for the study of soil-tool interaction [45–47]. We assigned to the particles the typical mechanical properties of sand, while the choice of the microscale contact parameters is arbitrary and in the usual range of values employed in DEM studies. The probes were modelled as rigid bodies, thus neglecting elastic deformations. The chosen penetration speed (*v* = 10 mm/s) is higher than the experimental value in order to reduce the duration of the simulations, since the total penetration force can be assumed to be nearly independent from *v* for small penetration velocities [42]. Additional details on the DEM simulations are reported in the Supporting Information (S2 and S3 Figs, S1 Table, S1 Movie, S1 Note). The study of the soil dynamics, which is anyway of little importance for quasi-static penetrations as in our case, is not an objective of the present work since it is not expected to change the qualitative performance and the differences among the considered probe geometries.

## Results

The 3-day old *Zea mays* roots, grown in air, showed a length of 81.62±10.12 mm, a tip diameter of 0.88±0.1 mm, and a root diameter of 1.19±0.1 mm (refer to Fig 2 for the definition of these physical features). The characteristic tip ratio is equal to 1.12 ± 0.07 (refer to material and methods section for more details on the definition of these physical features).

After a fitting analysis, the function that better replicates the root tip morphology was:

$$F\left(x\right)=ax^{b}$$ (2)

with coefficients, a = 2040, b = -0.59 (r-square = 0.9974).

**Fig 6:**
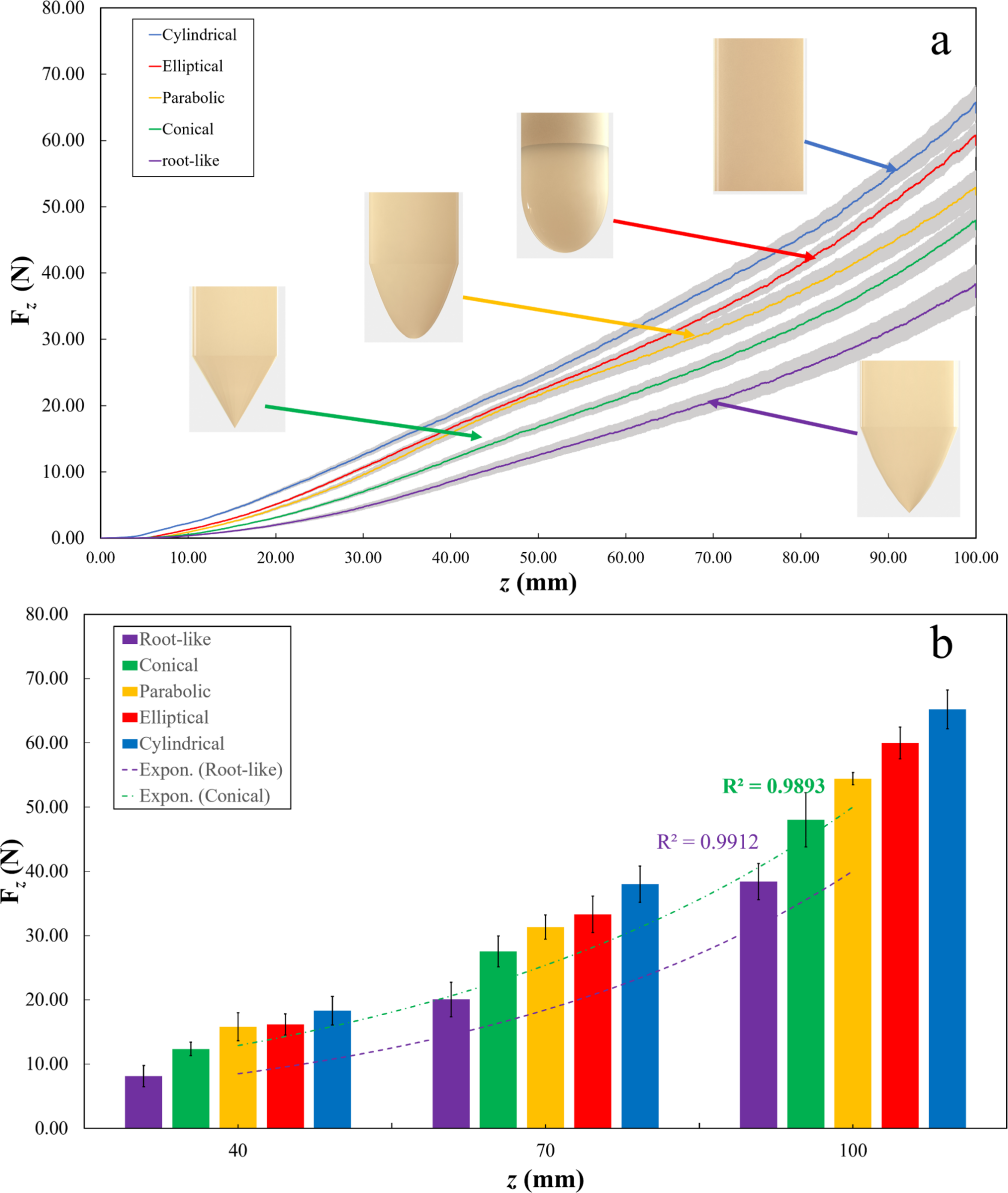
Soil penetration test of five different probes. (a) Force profile, a diameter of the tested probes is equal to 7 mm and the maximum reached penetration depth was 100 mm at 10 mm/min speed; (b) Force comparison at different depths.

This mathematical function, together with the characteristic tip ratio, was used to design the CAD model of the bioinspired probe. Fig 6A shows a comparison of force penetration among different tip shapes with 7 mm diameter. During soil penetration, the graphs showed that the root-like probe needs 72%, 59%, 44%, and 30% force less than the cylindrical, elliptical, parabolic, and conical tips, respectively. The cylindrical probe showed the highest measured forces for all the different diameters, ranging from 96.18 N to 8.28 N for probe diameters ranging from 11 mm to 3 mm, respectively (characterization of all the diameters curves are reported in S4, S5, S6 and S7 Figs). The root-like probe showed the lowest force for all the diameter probes tested, ranging from 71.98 N to 6.02 N for probe diameters ranging from 11 mm to 3 mm of diameter probe, respectively. Table 1 summarizes the measured force values gathered by pushing the probes up to 100 mm penetration depth with different tips and diameters. Fig 6B shows the force at different depths (40 mm, 70 mm, and 100 mm) exponentially increasing over depth (a comparison of all probes with different diameters at different depths are reported in S8, S9, S10 and S11 Figs). Moreover, we observed that penetration force linearly increases with the probe diameter (S12 Fig).

**Table 1:** Penetration force (mean ±SD) (N) of artificial probes in correspondence of 100 mm penetration depth.

Probe diameter	Tip shape				
	Cylinder	Ellipse	Parabola	Cone	Root-like
11 mm	96.18±4.58	88.96±4.48	87.59±2.80	76.23±4.05	71.98±5.83
9 mm	85.18±5.01	70.26±2.50	67.41±1.18	62.15±5.51	57.00±6.27
7 mm	65.21±3.01	59.98±1.40	54.53±0.90	49.33±5.03	37.84±2.86
5 mm	26.20±2.24	23.42±1.83	21.71±1.23	20.53±1.09	18.94±1.64
3 mm	8.28±0.81	7.430±0.67	6.420±0.63	6.230±0.90	6.020±0.75

**ANOVA analysis was carried out (the results are collected in Table 2) to compare the gathered data. A statistically significant difference (p**-**value < 0.05) was found among the five artificial probes in correspondence of all the probe diameters.**

**Table 2:** ANOVA test results of different artificial probes.

Probe diameter	ANOVA Test variables			
	DF	F	F-critical	P-value
11 mm	(4,19)	18.86	2.89	2.08E-06
9 mm	(4,21)	21.17	2.84	4.02E-07
7 mm	(4,27)	61.78	2.73	3.39E-13
5 mm	(4,20)	11.28	2.87	5.89E-05
3 mm	(4,24)	7.43	2.78	0.000483

A measurement of the work needed for the penetration was also calculated as follows:

E=∫F(s)ds (3)

Table 3 collects the average work expended by the probes with different tips and diameters to reach 100 mm penetration depth. As observed for force comparison, the cylindrical tip showed the worst performance in terms of expended work, ranging from 4.27 J to 0.36 J for probe diameters ranging from 11 mm to 3 mm, respectively. Also, from an energetic point of view, the root-like probe expended the lowest work for penetration, ranging from 2.91 J to 0.26 J for probe diameters ranging from 11 mm to 3 mm, respectively. Comparing the different probes, for example, in the case of 7 mm diameter, root-like probe expended 89%, 67%, 54%, and 35% less work than the cylindrical, elliptical, parabolic, and conical probe, respectively.

**Table 3:** Expended penetration work (mean ±SD) (J) of artificial probes up to 100 mm penetration depth.

Probe diameter	Tip shape				
	Cylinder	Ellipse	Parabola	Cone	Root-like
11 mm	4.27±0.20	3.63±0.20	3.53±0.10	3.05±0.04	2.91±0.10
9 mm	3.72±0.22	2.76±0.13	2.65±0.11	2.35±0.28	2.23±0.23
7 mm	2.65±0.21	2.34±0.17	2.16±0.18	1.89±0.18	1.40±0.18
5 mm	1.13±0.11	0.95±0.09	0.92±0.07	0.87±0.08	0.74±0.04
3 mm	0.36±0.03	0.34±0.01	0.31±0.02	0.28±0.04	0.26±0.01

**Fig 7:**
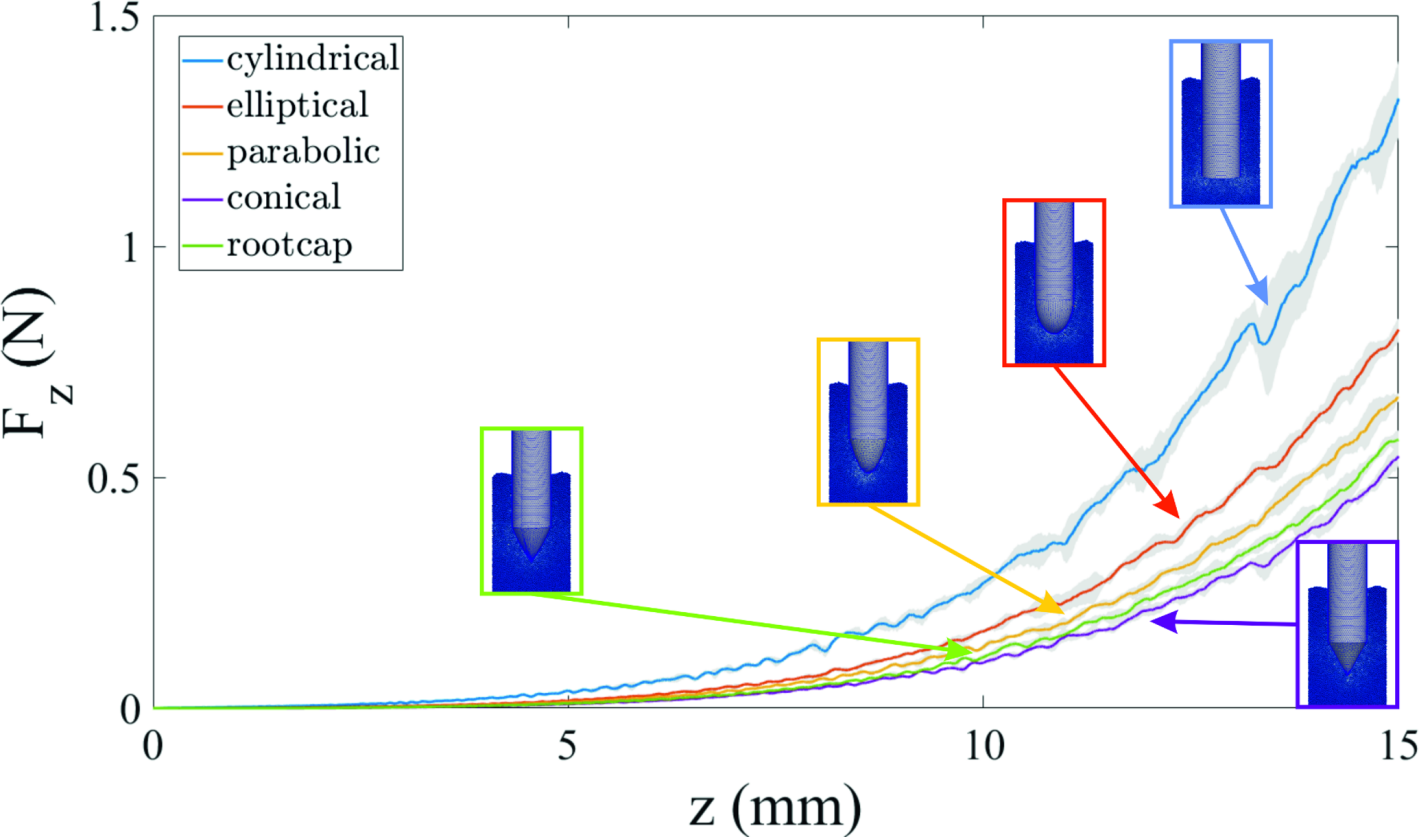
Soil penetration curves of five different probes from numerical simulations. The diameter of the tested probes is equal to 3 mm and the maximum reached penetration depth was 15 mm at 10 mm/s speed. The lines refer to the average values of five random simulations, the shaded areas to the standard deviations.

Fig 7 presents the penetration curves, obtained by numerical simulations, of the considered probes up to 15 mm penetration depth. The results are in good agreement with the experimental data, which return a higher penetration force for the cylindrical, elliptical and parabolic probes, in this order, while the conical and root-like probes present the lowest values.

The penetration depth shown in Fig 7 can be corrected to take into account the effect of the lateral walls: then the soil penetration curves can be rescaled as explained in S1 Note, S4, S5, S6, S7 and S12 Figs.

**Fig 8:**
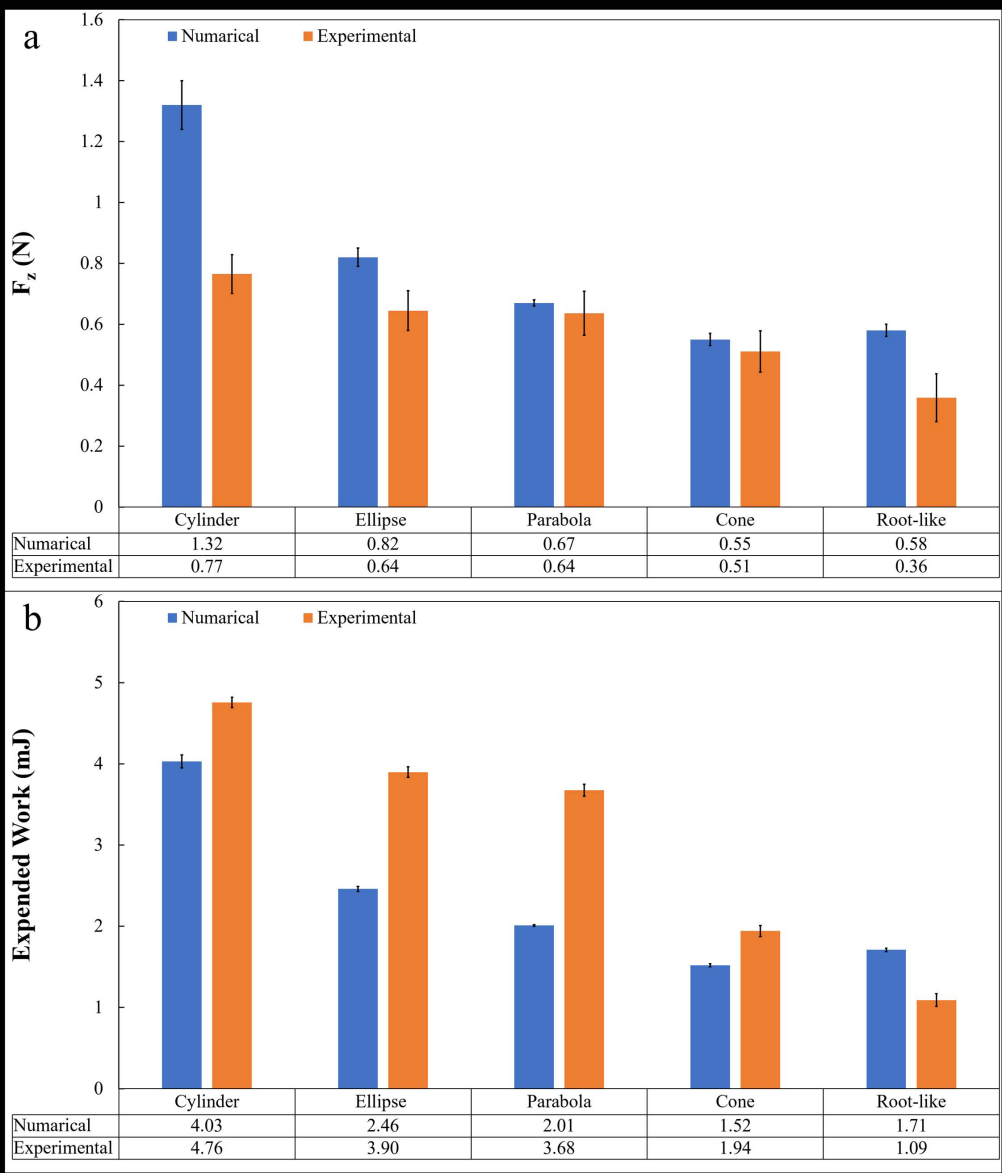
Comparison between numerical and experimental results. (a) Penetration force (mean ± SD) (N); (b) Expended penetration work (mean ± SD) (mJ) from numerical simulations of 3 mm probes in correspondence of 15 mm penetration depth.

Fig 8 reports the numerical values of the penetration force and the expended penetration work at 15 mm penetration depth, compared with the experimental data. Except for the parabolic probe, also the simulated values of the penetration work are in good agreement with those observed experimentally and fall in the same order of magnitude. Differences can be explained in the approximations introduced through numerical simulations, above all the description of the soil with spherical particles and the arbitrary values assigned to the microscale contact parameters, which can be tuned adequately to compensate the different considered volume.

## Discussion

The present work aims at highlighting how the tip morphology of a penetrometer can affect its performance in real soil penetration. In particular, our study proves that an artificial probe with a plant root-like shape is more efficient in terms of penetration force and expended work compared with standard shape probes (cylindrical, parabolic, elliptical, or conical tip) at different sizes. Among the considered tip morphologies, as we expected, cylindrical probes showed, for all the five diameters tested, the worst performance. The root-like probes showed less penetration force, namely 34%, 49%, 72%, 38% and 37% with respect to a cylindrical tip in correspondence of 11 mm, 9 mm, 7 mm, 5 mm, and 3 mm of probe diameter. Similarly, the root-like probes expended less work, namely 46%, 67%, 89%, 52%, and 38% in comparison with the cylindrical probes. Moreover, with the study of different diameters of the probe, we concluded that it varies linearly and could be used to select the probe size and shape for designing the robot for soil exploration according to soil types. Despite these good performances, selected material for probe showed buckling behaviours for smaller diameters (≤3 mm). Further studies will be dedicated to unveiling the importance of materials in these penetration tasks and to identify materials that are not affected by buckling behaviour. With this study, we demonstrated that the role of tip morphology is crucial for the penetration efficiency and provide an important cue of inspiration for future penetration systems. Also, our numerical simulations showed good agreement with the experimental data. Although the numerical simulations were not able to discriminate between conical and root-like probes because of the very small geometric difference and the considered particle diameter, the main result that the conical and root-like shapes are the optimal ones is retrieved.

Moreover, we found that the geometries with higher penetration forces present also higher values of the average contact number, i.e. the number of particles simultaneously in contact with a given central particle (see S3 Fig). This result suggests that the local density of the granular medium (which can be related to the average contact number) might be one of the key parameters controlling the penetration process. Therefore, the numerical simulations are potentially a useful tool for designing and optimizing new bio-inspired probe geometries. Future research work in this direction should be addressed to the determination of the optimal parameters for simulating penetration mechanics in granular matter.

Furthermore, the methodological approach described in the present paper can encourage a new research trend in the investigation of a possible correlation between root tip morphology and different plants habitat. In this study, we investigated *Z*. *mays*, one of the three most important cereal crops together with wheat and rice. *Z*. *mays* can be found all over the world for its capability to grow in well-drained light (sandy), medium (loamy), and heavy (clay) soils. This versatility represents the first reason for which we have focused our studies on this plant. Its exceptional capability to penetrate various kinds of soil can, in fact, represent an amazing source of inspiration for artificial prototypes. Nevertheless, further studies on different plants, living in a different habitat, are required to shed light on the possibility to find *ad hoc* tip morphologies for each kind of soil and can provide exceptional design cues for developing innovative artificial prototypes. Our findings can also contribute, beyond to the probe design benchmarks, to open discussion on the energetic efficiency issue in soil penetration, not still completely understood. Most of the existing studies in this field refer to the analysis of a projectile penetration in a solid medium: different geometries of the projectile have been investigated using empirical and semi-empirical models to predict the penetration depth [48–50]. Some other studies have mainly been focused on the energetic analysis during static or dynamic penetration tests in geological strata. Guirgis *et al*. [51] have proposed an energy approach to study the penetration and an energy transfer to the rod by hammer using SPT. CPT or other dynamic penetration test methods have been investigated by other researchers [52–56]. Among all these studies, none has ever investigated the morphological features of plant roots in the penetration tasks.

## Supporting information

S1 Fig Soil type. Soil texture triangle, which was characterized for the experimental purpose.
(DOCX)

S2 Fig Simulated probe geometries immersed in the granular packing. From left to right: cylinder, ellipse, parabola, cone and root-like.
(DOCX)

S3 Fig Contact number curves of five different probes from numerical simulations. The diameter of the tested probes is equal to 3 mm and the maximum reached penetration depth was 15 mm at 10 mm/s speed. The lines refer to the average values of five random simulations, the shaded areas to the standard deviations.
(DOCX)

S1 Table Material properties and main DEM simulation settings. (DOCX)

S1 Note Effective penetration depth for DEM numerical simulations: Explanation of numerical simulation model. (DOCX)

S1 Movie Numerical simulation with different morphologies. Sample simulation video of probes with different morphologies such as cylindrical, elliptical, parabolic, conical and root-like. The simulation is based on numerical method (Dynamic Element method) in cylindrically bounded soil environment.
(MP4)

S4 Fig Soil penetration curves of five different shapes for 11 mm diameter probe. Maximum reached penetration depth was 100 mm at 10 mm/min speed.
(DOCX)

S5 Fig Soil penetration curves of five different shapes for 9 mm diameter probe. Maximum reached penetration depth was 100 mm at 10 mm/min speed.
(DOCX)

S6 Fig Soil penetration curves of five different shapes for 5 mm diameter probe. Maximum reached penetration depth was 100 mm at 10 mm/min speed.
(DOCX)

S7 Fig Soil penetration curves of five different shapes for 3 mm diameter probe. Maximum reached penetration depth was 100 mm at 10 mm/min speed.
(DOCX)

S8 Fig Comparison of penetration force at different depths of five different shapes for 11 mm diameter probe. Maximum reached penetration depth was 100 mm at 10 mm/min speed.
(DOCX)

S9 Fig Comparison of penetration force at different depths of five different shapes for 9 mm diameter probe. Maximum reached penetration depth was 100 mm at 10 mm/min speed.
(DOCX)

S10 Fig Comparison of penetration force at different depths of five different shapes for 5 mm diameter probe. Maximum reached penetration depth was 100 mm at 10 mm/min speed.
(DOCX)

S11 Fig Comparison of penetration force at different depths of five different shapes for 3 mm diameter probe. Maximum reached penetration depth was 100 mm at 10 mm/min speed.
(DOCX)

S12 Fig Penetration force change over different diameters and depths of the probes. (DOCX)
