# Supplementary material for: A study on plant root apex morphology as a model for soft robots moving in soil
Source: PLoS One. 2018 Jun 6;13(6):e0197411. doi: 10.1371/journal.pone.0197411 (PMC5991344; doi:10.1371/journal.pone.0197411)
Supplement: S1 Fig — Soil texture triangle, which was characterized for the experimental purpose. (DOCX) [file pone.0197411.s001.docx]

**
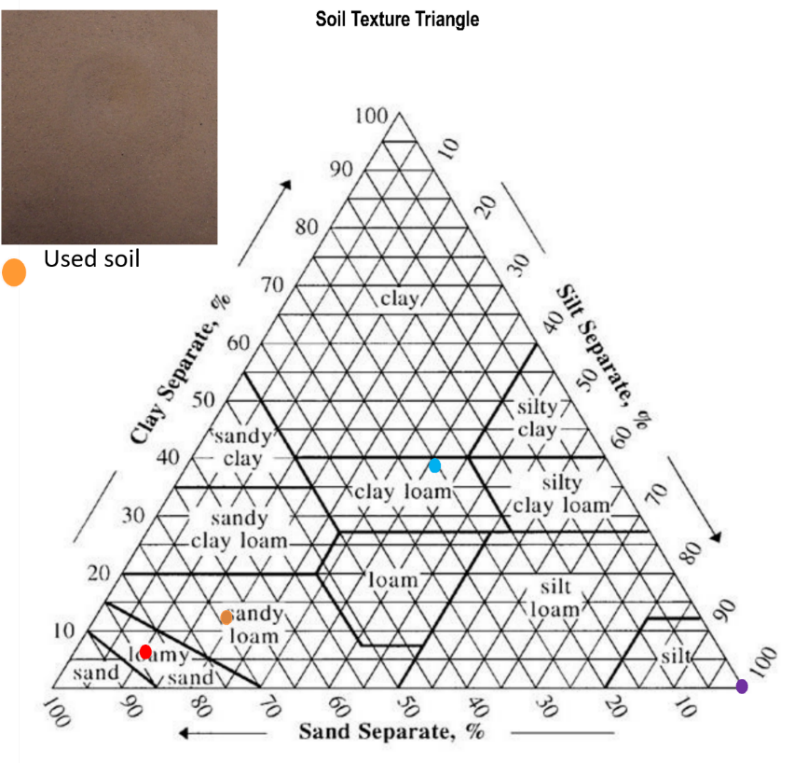
**

**S1 Fig. Soil type and characterization.** Soil texture triangle which is characterized for the experimental purpose
