# Supplementary material for: A study on plant root apex morphology as a model for soft robots moving in soil
Source: PLoS One. 2018 Jun 6;13(6):e0197411. doi: 10.1371/journal.pone.0197411 (PMC5991344; doi:10.1371/journal.pone.0197411)
Supplement: S2 Fig — From left to right: cylinder, ellipse, parabola, cone and root-like. (DOCX) [file pone.0197411.s002.docx]

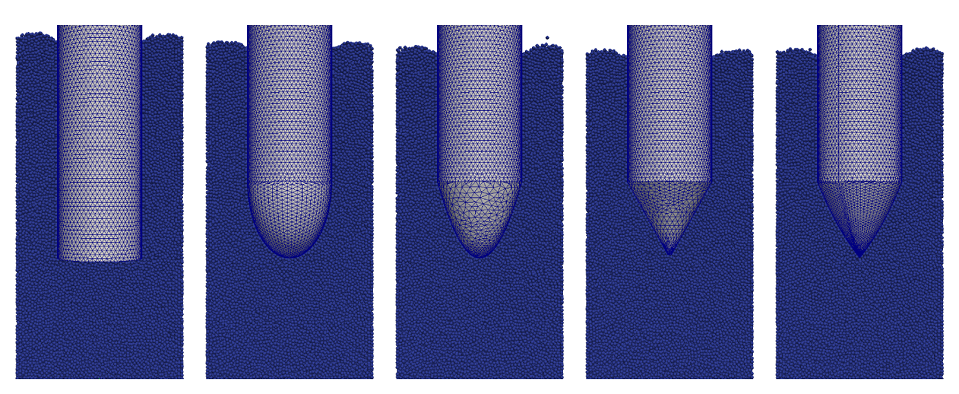


**S2 Fig. Simulated probe geometries immersed in the granular packing.** From left to right: cylinder, ellipse, parabola, cone and root-like.
