# Supplementary material for: A study on plant root apex morphology as a model for soft robots moving in soil
Source: PLoS One. 2018 Jun 6;13(6):e0197411. doi: 10.1371/journal.pone.0197411 (PMC5991344; doi:10.1371/journal.pone.0197411)
Supplement: S3 Fig — The diameter of the tested probes is equal to 3 mm and the maximum reached penetration depth was 15 mm at 10 mm/s speed. The lines refer to the average values of five random simulations, the shaded areas to the standard deviations. (DOCX) [file pone.0197411.s003.docx]

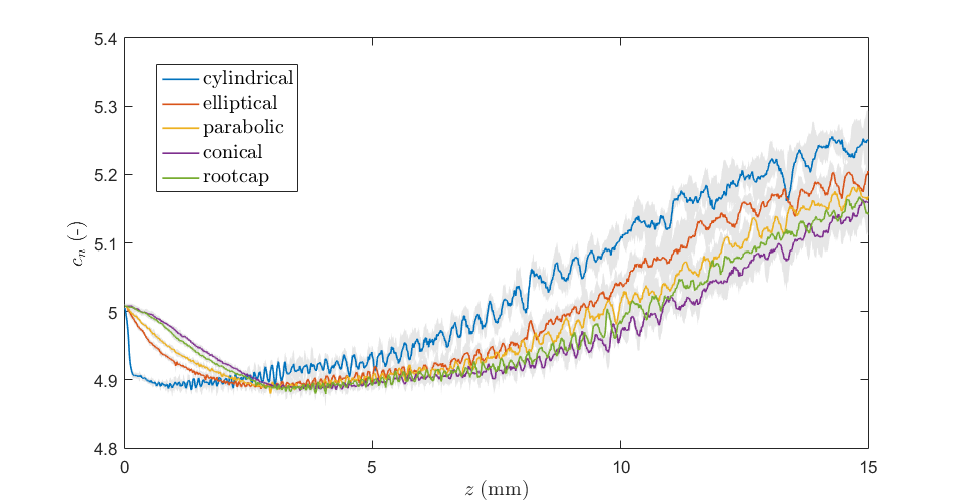

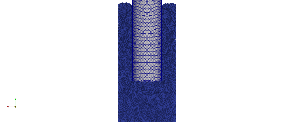

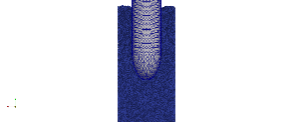

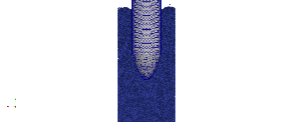

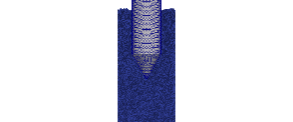

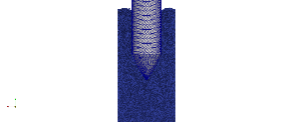


S3 Fig. Contact number curves of five different probes from numerical simulations. The diameter of the tested probes is equal to 3 mm and the maximum reached penetration depth was 15 mm at 10 mm/s speed. The lines refer to the average values of five random simulations, the shaded areas to the standard deviations.
