# Supplementary material for: A study on plant root apex morphology as a model for soft robots moving in soil
Source: PLoS One. 2018 Jun 6;13(6):e0197411. doi: 10.1371/journal.pone.0197411 (PMC5991344; doi:10.1371/journal.pone.0197411)
Supplement: S1 Table — (DOCX) [file pone.0197411.s004.docx]

**S1Table.** Material properties and main DEM simulation settings.

| **Property** | **Value** |
| --- | --- |
| sand density | 2000 kg/m^3^ |
| sand Young’s modulus | 10 MPa |
| sand Poisson’s ratio | 0.3 |
| probe Young’s modulus | 1 GPa |
| probe Poisson’s ratio | 0.3 |
| sand-sand coefficient of friction | 0.5 |
| sand-probe coefficient of friction | 0.5 |
| sand-sand coefficient of restitution | 0.8 |
| sand-probe coefficient of restitution | 0.8 |
| particle diameter | 0.125 mm |
| number of particles | 40000 |
| penetration velocity | 10 mm/s |
| time step | 1∙10^-6^ s |
