# Supplementary material for: A study on plant root apex morphology as a model for soft robots moving in soil
Source: PLoS One. 2018 Jun 6;13(6):e0197411. doi: 10.1371/journal.pone.0197411 (PMC5991344; doi:10.1371/journal.pone.0197411)
Supplement: S1 Note — (DOCX) [file pone.0197411.s005.docx]

**S1 Note. Effective penetration depth for DEM numerical simulations**

The force - displacement curves obtained through the numerical simulations must be corrected in order to take into account the effective penetration depth of the probe. The presence of the lateral walls, in fact, has the effect of a progressive increase in the height of the particle packing, since particles are displaced from the volume occupied by the probe during penetration.

The volume occupied by the probe *V_p_* changes as function of time as:

$$V_{p}\left(t\right)=\left\{\begin{matrix} \chi\pi R_{p}^{2} v t v t\leq H_{p} \\ \pi R_{p}^{2} v t+\pi R_{p}^{2} H_{p} \left(\chi-1\right) v t>H_{p} \end{matrix}\right.$$

where *R_p_* is the probe radius, *H_p_* the tip height and *v* the penetration speed. *χ* is a shape factor depending on the probe geometry:

$$\chi=\frac{1}{H_{p }R^{2}} \int_{0}^{H_{p}} \left(f^{-1}\left(y\right)\right)^{2}\text{d}y$$

and $\frac{1}{3}\leq\chi\leq1$with the lowest limit corresponding to the conical probe (i.e., minimum tip volume) and $\chi=1$to the cylindrical probe (i.e., maximum tip volume).

If *R* is the container radius and *H* the height of the particle packing, its variation during the penetration process is given by:

$$\pi\left(R^{2}-R_{p}^{2}\right) \cdot\Delta H\left(t\right)\approx V_{p}\left(t\right)$$

Therefore, the probe displacement $z\left(t\right)=v t$ can be corrected by adding$\Delta H\left(t\right)$, obtaining the following expression for the effective probe displacement:

$$z_{\text{eff}}\left(t\right)\approx\left\{\begin{matrix} v t \left(1+\frac{R_{p}^{2}}{R^{2}-R_{p}^{2}} \chi\right) v t\leq H_{p} \\ v t+\frac{R_{p}^{2}}{R^{2}-R_{p}^{2}} \left[v t+H_{p} \left(\chi-1\right)\right] v t>H_{p} \end{matrix}\right.$$

Note that in the case of the experimental tests, the proposed displacement correction is negligible since $\frac{R_{p}}{R}<<1$.

The rescaling of the penetration depth according to the expression above produces lower values of the penetration force. This effect could be compensated by the assumed values for some microscale parameters of the DEM simulations (e.g., the coefficients of friction and of restitution listed in Table S1), which can be tuned to match precisely the experiments. Since the order relations among the different tip shapes is preserved, here we have considered the nominal penetration depth without the rescaling above derived.

The videos show a part of the penetration process for the considered probe geometries, together with the measured particle-particle interaction forces (in units of 1∙10^-9^ N). For visualization purposes, only half of the particle packing is shown, and the videos are four times faster than the applied penetration velocity (See S1 Movie).
