# Supplementary material for: A study on plant root apex morphology as a model for soft robots moving in soil
Source: PLoS One. 2018 Jun 6;13(6):e0197411. doi: 10.1371/journal.pone.0197411 (PMC5991344; doi:10.1371/journal.pone.0197411)
Supplement: S6 Fig — Maximum reached penetration depth was 100 mm at 10 mm/min speed. (DOCX) [file pone.0197411.s009.docx]

**
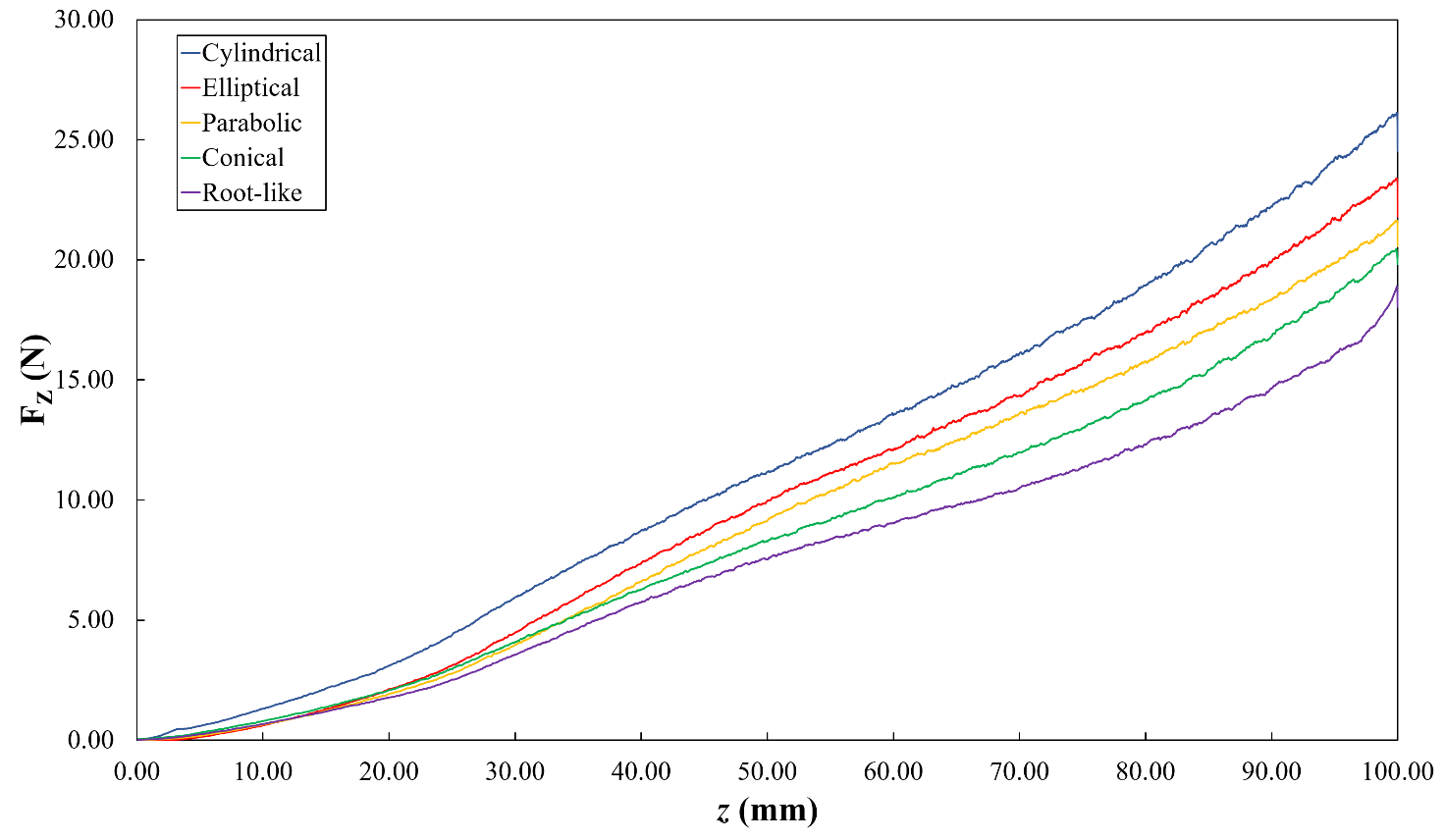
**

**S6 Fig. Soil penetration curves of five different shapes for 5 mm diameter probe.** Maximum reached penetration depth was 100 mm at 10 mm/min speed
