# Supplementary figures and images for: A study on plant root apex morphology as a model for soft robots moving in soil
Source: PLoS One. 2018 Jun 6;13(6):e0197411. doi: 10.1371/journal.pone.0197411 (PMC5991344; doi:10.1371/journal.pone.0197411)

**
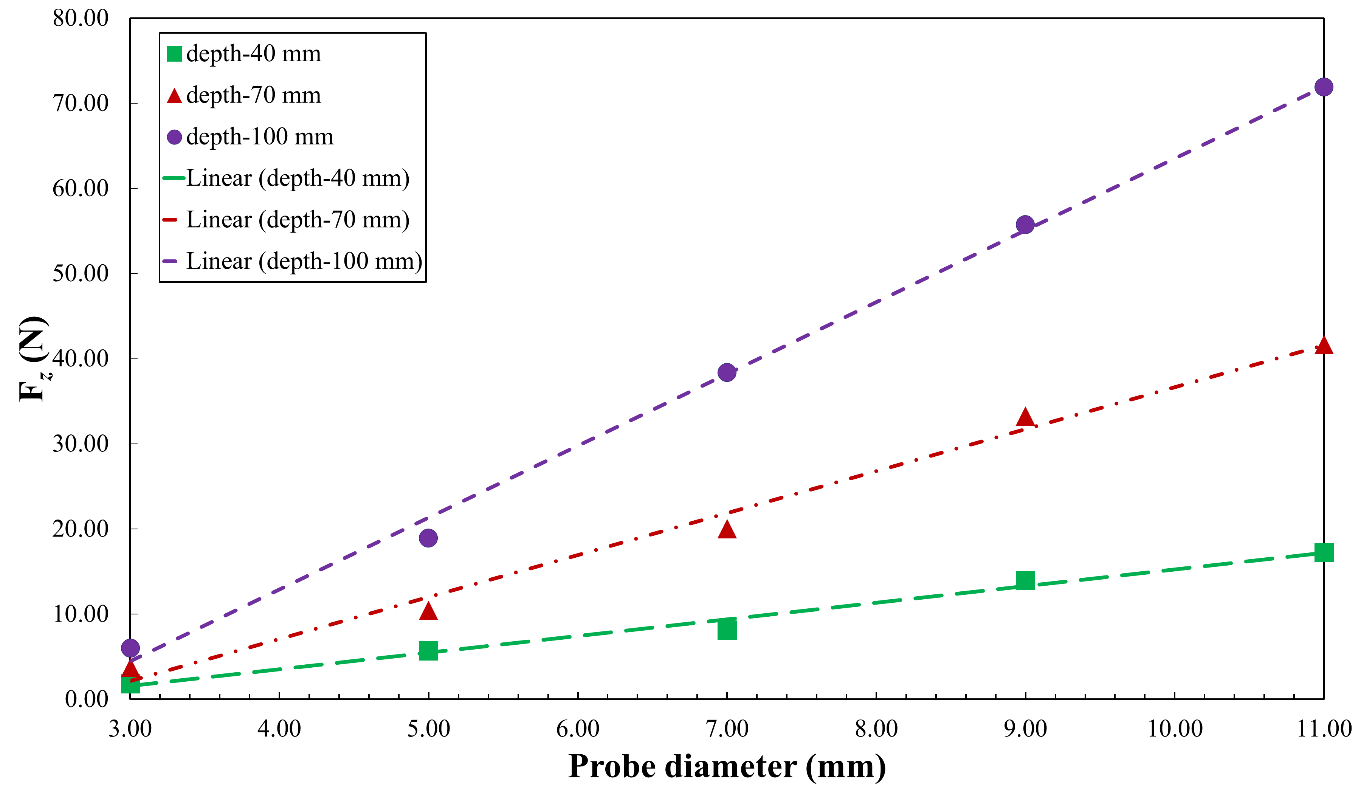
**

**S12 Fig. Penetration force change over different diameters and depths of the probes**

Supplement: S12 Fig — (DOCX) [file pone.0197411.s015.docx]
